# A Hadoop-Based Platform for Patient Classification and Disease Diagnosis in Healthcare Applications

**DOI:** 10.3390/s20071931

**Published:** 2020-03-30

**Authors:** Hassan Harb, Hussein Mroue, Ali Mansour, Abbass Nasser, Eduardo Motta Cruz

**Affiliations:** 1 ICCS-Lab, American University of Culture and Education (AUCE), Beirut 1105, Lebanon; abbassnasser@auce.edu.lb; 2 Lab-STICC, CNRS UMR 6285, Ensta-Bretagne, 29200 Brest, France; mansour@ieee.org; 3 Institute of Electronics and Telecommunications of Rennes, University of Nantes, CNRS, IETR UMRS 6164, 85000 La Roche-sur-Yon, France; hussein.mroue@univ-nantes.fr (H.M.); Eduardo.Mottacruz@univ-nantes.fr (E.M.C.)

**Keywords:** healthcare applications, hadoop platform, patient classification, disease diagnosis, SK-means, association mining rules

## Abstract

Nowadays, the increasing number of patients accompanied with the emergence of new symptoms and diseases makes heath monitoring and assessment a complicated task for medical staff and hospitals. Indeed, the processing of big and heterogeneous data collected by biomedical sensors along with the need of patients’ classification and disease diagnosis become major challenges for several health-based sensing applications. Thus, the combination between remote sensing devices and the big data technologies have been proven as an efficient and low cost solution for healthcare applications. In this paper, we propose a robust big data analytics platform for real time patient monitoring and decision making to help both hospital and medical staff. The proposed platform relies on big data technologies and data analysis techniques and consists of four layers: real time patient monitoring, real time decision and data storage, patient classification and disease diagnosis, and data retrieval and visualization. To evaluate the performance of our platform, we implemented our platform based on the Hadoop ecosystem and we applied the proposed algorithms over real health data. The obtained results show the effectiveness of our platform in terms of efficiently performing patient classification and disease diagnosis in healthcare applications.

## 1. Introduction

Today, the world faces an increasing number of diseases and patients. In addition, wars, pollution, food-related illness, and human–animal relationships cause the emergence and propagation of new types of diseases and viruses. Subsequently, with the emergence of an unknown disease, the governments face two major challenges: first, medical centers require more and more qualified staff in order to periodically monitor each patient and quickly act when an emergency state is detected. Second, the correct diagnosis of the patient’s state and the progress of his situation are critical for medical staff. Recently, the “Coronavirus” propagated in China has threatened millions of people and the government was obliged to quickly build hospitals and increase the number of medical workers. Furthermore, integrating new technologies in healthcare has became an essential task in order to make hospitals more efficient in terms of monitoring and analyzing patient data, thus enhancing medical processes.

Recently, the emergence of sensing-based devices, especially wireless sensor network (WSN) and Internet of Things (IoT), and big data technologies, such as Hadoop ecosystem, lead to a new revolution in healthcare. Basically, the sensing-based healthcare consists of a set of biomedical sensors that allows continuously monitoring the vital signs (e.g., heart rate, respiration rate, oxygen rate, temperature, blood pressure, etc.) of a patient and periodically transmit the collected data to a coordinator for further inspection. Smart technologies have opened up a world of applications in disease diagnostic and treatment such as for cancer, glucose monitoring, depression, Parkinson’s disease, connected contact lenses, etc. Furthermore, this has been a big motivation for healthcare organizations to heavily invest in big data analytics.

However, sensing-based healthcare applications are still challenging the scientific community due to:

- Big data collection and storage: Biosensors continuously record vital signs of patients, usually per second, and then they send their records toward data storage center. This type of data has three characteristics: (1) massive data collection; (2) high speed generation; and (3) heterogeneous nature. Unfortunately, the relational database management systems and the traditional data storage technologies cannot handle such type and amount of data.
- Rapid emergency detection: Usually, a normal health range is defined for each vital sign. Records outside this range lead to an abnormal situation which should be quickly detected and reported to the medical staff in order to take suitable actions. Hence, rapid emergency detection is a crucial event that can be a threat to patient safety or even life.
- Disease diagnosis and patients’ classification: In health applications, several diseases may generate similar symptoms. Hence, disease diagnosis is susceptible to human error which may provoke disabilities or death. Therefore, developing new techniques and algorithms in order to study, on the one hand, the correlation between symptoms and, on the other hand, classify patients according to their situations can help in making the right decision.

To overcome the above challenges, we propose an efficient and robust big data analytical platform for real-time sensing-based healthcare applications. The proposed platform relies on big data technologies, specifically Hadoop ecosystems, and it uses data analytical techniques for data analysis and classification. In addition, our platform consists of four layers: real time patient monitoring, real time decision and data storage, patient classification and disease diagnosis, and data retrieval and visualization. We conducted a set of scenarios and simulations on real health data in order to show the relevance of our platform.

The remainder of this paper is organized as follows. Section 2 presents an overview on various existing systems in the literature. In Section 3, we introduce the architecture of our proposed platform. Section 4, Section 5, Section 6 and Section 7 detail tools and proposed algorithms in each layer, respectively. Section 8 describes the implementation of our platform and explains the obtained results. Finally, Section 9 concludes the paper and gives our directions for future work.

## 2. Related Work

Recently, remote sensing and Hadoop ecosystem have produced efficient and low cost solutions in various domains, especially in healthcare applications. On the one hand, sensing devices help doctors and nurses to remotely, in-hospital or in-home, monitor patients and allow a real-time detection of urgent situations. On the other hand, Hadoop-based tools offer an efficient and rapid data storage and processing platforms for hospitals, especially in specific scenarios such as war or global virus contamination. Therefore, researchers have proposed various healthcare systems based on remote sensing and Hadoop dedicated to disease diagnosis, emergency detection, patient classification, etc. [1,2,3]. In [4,5], the authors gave an overview on different data analytical algorithms and big data platforms proposed in the literature for healthcare applications.

Some works in sensing-based healthcare are focused on reducing big data collection using aggregation, compression, and prediction methods [6,7,8,9,10,11,12]. The authors of [6] proposed the Priority-based Compressed Data Aggregation (PCDA) technique to reduce the amount of heath data transmitted. PCDA uses a compressed sensing approach followed by a cryptographic hash algorithm to save information accuracy before sending data for any diagnosis. The authors of [7] proposed a data reduction technique dedicated to wireless seizure systems. In addition to local compressive sensing, the proposed technique selects a set of features, specifically those with nonlinear autocorrelation, to reduce the seizure signals sent to the data server. In [8], a cloud-based connected healthcare system, called BigReduce, is proposed. The objective of BigReduce is to minimize the data processing cost at the base station according to two schemes applied locally at the IoT sensors: reduction and decision schemes. Finally, the authors of [9] proposed a fault tolerance and data recovery algorithm in order to ensure the integrity of heath information and save the network energy. The proposed algorithm works in a distributed manner and it maintains a set of active nodes where each one copies its data to its neighbors according to several assumptions.

Other works in sensing-based healthcare are based on Hadoop ecosystems in order to efficiency managing data collected in such network [13,14,15,16,17,18]. The authors of [13] proposed a storage and processing architecture based on MapReduce and HBase, respectively. First, data generated by health sensors are inserted into HBase using a mass insertion script, and then a data analysis algorithm is proposed in order to retrieve valuable data and help in predicting disease. In [14], the authors proposed a Hadoop-based framework in order to secure the transmitted data from biosensors to the server. The proposed framework relies on three main pillars: The Hadoop clusters for storing, backing up, and recovering data; a digital signature method, called ECC (Elliptic Curve Cryptography), which is implemented in Sunspot nodes in order to secure communication; and the Sqoop tool, which is used to import and export data between Mysql and Hadoop HDFS. In [15], a modern platform for healthcare information systems consisting of three layers is proposed. The first layer is composed of various data health sources such as sensors, clinical report, medication, etc. The second layer aims to process and store data and it uses various Hadoop tools including Sqoop, HDFS, HBase, MapReduce. and Hive. The last layer is responsible for applying business intelligence (BI) solutions over the stored data and it uses SpagoBI tools as an open source BI suite. Lastly, the authors of [16] proposed a correlation-based outlier detection platform, relying on Hadoop, to handle streaming of data generated in health applications. The objective of the platform is to search statistical relationships among biosensors, and then, in a later step, to predict contextual anomalies using an aggregated outlier checker.

Recently, the authors of [19,20,21,22] opened a new trend in sensing-based healthcare by proposing several frameworks for real-time patient monitoring and assessment. In [19], a framework for stress detection and evaluation is proposed. The framework works by detecting first stress signals according to skin conductance parameter, and then the stress level is evaluated through fuzzy inference system based on patient vital signs, particularly heart rate, respiration rate, and average blood pressure. In a later work [20], the authors proposed a data management framework for data collection and decision making in sensing-based healthcare. The framework relies on three algorithms: first, an emergency detection algorithm aims to send critical records directly to the coordinator; second, an adaptive sampling rate algorithm based on ANOVA (ANalysis of Variance) and Fisher test allows each sensor to adapt its sampling to the variation of patient situation; and third, a data fusion and decision making model is proposed to the coordinator based on decision matrix and fuzzy set theory. Despite its great advantages for patient monitoring and assessment, the proposed framework suffers from several disadvantages: (1) sensors only send critical records to the coordinator, thus medical staff cannot avoid patients entering dangerous situations before this happens; (2) in case of low critical patients, none of the data would be archived in the hospital, thus revising patient archives to check patient progress from doctors is not possible; and (3) integrating new data technologies, such as Hadoop, in order to handle big data collected by biosensors is not considered in the proposed framework. To the best of our knowledge, our proposed platform is still the only complete system that integrates both data analytics and Hadoop ecosystems. In addition, it consists of a sequence of layers aiming to tackle other problems related to patient classification and disease diagnosis. Finally, it combines several approaches such as machine learning, data mining, clustering, classification, etc.

## 3. Overview about the Architecture of Our Platform

In this section, we present an overview of the different tools and techniques forming the architecture of our platform. Mainly, our system relies on Hadoop ecosystems, which are built on clusters using parallel computing. On the one hand, the cluster architecture ensures a high scalability and reliability of the collected data as well as a fast and huge data storage. On the other hand, the parallel computing ensures a rapid data processing and analysis, especially when the volume of data becomes huge. Furthermore, the architecture of our platform consists of four layers, which allow an entire data analysis process for patients in hospitals, starting from patient data collection and ending with patients’ classification and decision making. Subsequently, each layer performs batch and/or real-time processing. In batch mode, data are first stored in cluster nodes and then processed, while, in real-time mode, data are processed upon collection and before storing. These two processing modes provide a high efficiency in terms of applying any desired techniques (machine learning, artificial intelligence, data mining, etc.) over the collected data.

**Figure 1:**
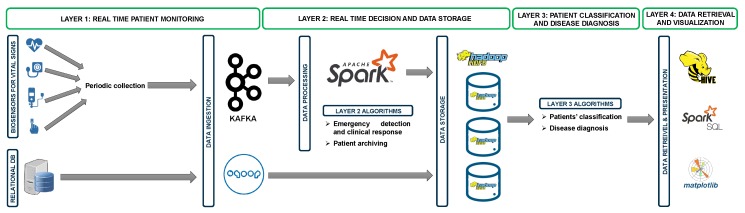
Architecture of our platform.

Figure 1 presents the architecture of our platform with all layers, which are briefly introduced as follows:

- Real time patient monitoring: This layer consists of data sources and data ingestion tools. Data sources are the active biosensors which allow continuously monitoring a patient and send data in real-time toward the data storage via data ingestion tools. In addition, data sources could be an archive of patients’ records which is already built by the hospital, mostly based on relational database system, and should also be sent toward the data storage center. We use two ingestion tools, namely kafka [23] and sqoop [24], to process and store streaming of data coming from different data sources.
- Real time data decision and data storage: This layer uses Spark [25] and Hadoop HDFS (Hadoop Distributed File System) [26] to process and store data, respectively. After installing it on the master cluster node, Spark receives data coming from Kafka and applies two algorithms: the first one allows detecting emergency situations and then taking the right action by the medical staff and the other searches for missing records before sending the final data to the HDFS for storage. Subsequently, data are stored in three Hadoop nodes according to HDFS cluster computing.
- Patient classification and disease diagnosis: This layer uses batch mode processing and aims to classify and find the correlation after data storing. Two algorithms are proposed: The first one uses an adapted version of Kmeans [27] clustering and aims to find patients with similar situations. The second one uses the data mining approach to allow better understanding when diagnosing any disease.
- Data retrieval and visualization: This layer allows medical staff to retrieve patient data from the data storage and then to visualize them in order to analyze and understand the patient’s situation. We use two tools from the Hadoop ecosystems (Hive [28] and SparkSQL [25]) and Matplotlib [29] from Python.

## 4. Layer 1: Real Time Patient Monitoring

Data collection is the first step in the healthcare lifecycle. The first layer aims to collect data coming from various data sources and sending them for a later storing. Mainly, it consists of data sources, Kafka, and Sqoop tools. In the following, we detail them.

### 4.1. Data Sources

In health applications, data collection is usually done through small biosensors implemented on the patient’s body in order to collect his vital signs. Subsequently, the vital signs of a patient include Heart Rate (HR), Systolic Blood Pressure (SBP), Respiration Rate (RR), Oxygen Saturations (OS), Body Temperature (BT), etc. Assume a set *P* of F patients as follows: $P=[P_{1},P_{2},\cdots ,P_{F}]$ where each patient p∈P is assigned various types of biosensors to a set *V* of vital signs, e.g., V=[HR,SBP,RR,OS,BT,⋯] with T indicating the number of observed vital signs in *V*. For analysis purposes, we assumed that the set of biosensors $B_{V}^{p}$, assigned to the patient *p*, periodically monitor the vital signs and then send the set of collected records ${}_{t}^{}R_{V}^{p}$ at period *t*, where ${}_{t}^{}R_{V}^{p}=\left[r_{1},r_{2},\cdots ,r_{T}\right]$ and $r_{i}$ is the collected reading of the vital sign $v_{i}\in V$, toward the coordinator.

Nowadays, saving an archive for patient vital signs during their hospitalization is a procedure in all hospitals. Indeed, Relational Datadase (RDB) is the most preferred archiving system for almost of them due to its simplicity and flexibility. Hence, to make it more efficient, our platform uses Sqoop (as explained in (Section 4.3) to allow hospitals to insert their old archives into Hadoop cluster nodes. However, unlike biosensors, which operate in real-time mode, Sqoop processes patient archives in batch mode.

### 4.2. Kafka

Kafka [23] is an open-source data ingestion tool used to handle massive real-time data. Today, it is used in many applications such as real-time monitoring of British gas smart home, matching driver and passenger at Uber, etc. Among other data ingestion tools, the selection of Kafka is motivated by: (1) its high horizontal scalability (up to hundreds of server nodes); (2) it can read data directly from the biosensors; (3) it decreases latency much than other tools; and (4) it is easy to be implemented and used thanks to built-in libraries in Python.

Generally, Kafka is based on the concept of “topics” and data are stored in the form of key-values. In our platform, we installed Kafka on the master cluster node (called broker) and we create a topic, named “Col2Proc”, allowing to read streaming data from biosensors then send them to further processing (e.g., Spark tool).

### 4.3. Sqoop

Sqoop [24] is a command line interface application used to transfer data between relational databases (RDB) and Hadoop nodes. Import and Export Sqoop are the major operations in Sqoop and used to transfer data from RDB to Hadoop and vice versa, respectively. In our platform, Sqoop is used in allow medical administrators to directly integrate their patients’ archive, supposed to be built with RDB, into Hadoop cluster nodes without any further processing operation. This service makes our platform more efficient and attractive for hospitals.

## 5. Layer 2: Real Time Data Decision and Data Storage

In this section, we introduce the second layer used in our platform, which is responsible for data processing and storing. This layer mainly relies on two tools, namely Apache Spark and Hadoop HDFS system, as well as it proposes two data processing algorithms: emergency detection and clinical response, and patient archiving.

### 5.1. Apache Spark

Spark [25] is an open-source cluster computing platform which is widely used for data processing in Hadoop ecosystems. In our framework, Spark is selected among other existing tools due to three major characteristics required in sensing-based healthcare: first, it supports both batch and streaming processing which are necessary to apply various data analytical algorithms; second, it ensures a lower latency level than other tools such as MapReduce, which is strongly required in health applications; and third it guarantees scalability to any number of cluster nodes required for the health application requirements. Therefore, in our platform, we implemented Spark on the master cluster node in order to receive data streaming from Kafka, perform processing, and send data to the Hadoop HDFS for storing purpose. Subsequently, data processing is done via two created scripts, one for the real time decision and other for patient archiving (explained below).

### 5.2. Hadoop HDFS

Hadoop HDFS [26] It is a distributed file system that stores data across a set of machines, known as cluster nodes. Hadoop HDFS splits received data into blocks, and then it distributes across the nodes. Generally, it is characterized by two aspects: First, it ensures high data reliability thanks to the replication process of data into nodes (at least three). This aspect is very useful in sensing-based healthcare so patient archives will never be lost if any hardware failure occurs. Second, data in Hadoop HDFS are treated in parallel computing so a high data retrieval process is ensured, which is necessary for heath application especially in emergency cases. In our platform, we created three cluster nodes while implementing Hadoop HDFS on each of them to store patient records in HDFS files.

### 5.3. Emergency Detection and Clinical Response Algorithm

After receiving periodic data sent from all biosensors, the coordinator should analyze them in real time and alert the medical staff in case of a patient emergency detection. In the second layer of our platform, we introduce an emergency detection algorithm that allows alerting in any abnormal situation of the patient as well as it determines the appropriate response the should be taken by the medical staff. To verify abnormal situations, let us first define the Early Warning Score (EWS) guide.

**Figure 2:**
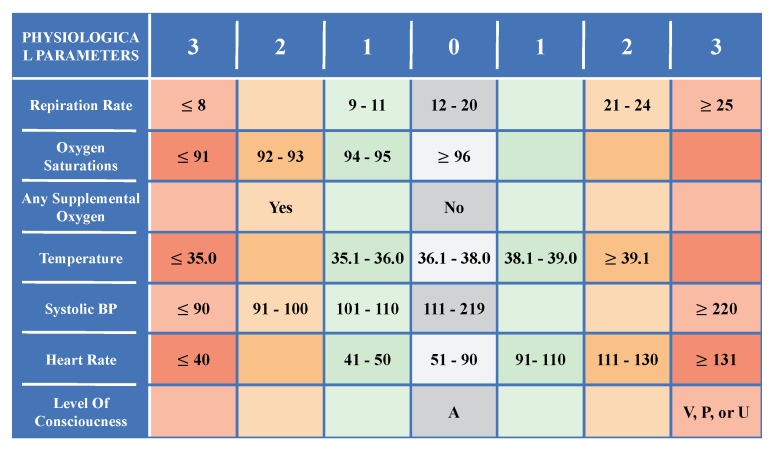
National Early Warning Score (NEWS) [30].

EWS is a guide based on the vital signs, e.g., *V*, and it is used by the medical staff within a hospital to track the criticality level of a patient. For each vital sign v∈V, the collected record $r_{i}\in {}_{t}^{}R_{V}^{p}$ is compared to a normal range in order to calculate a score $s_{i}$ between 0 and 3; 0 means normal record where other values indicate abnormal situations with increasing severity as the score increases. Hence, a set of record scores ${}_{t}^{}S_{V}^{p}=\left[s_{1},s_{2},\cdots ,s_{T}\right]$ is calculated for ${}_{t}^{}R_{V}^{p}$. In Figure 2, we show one of the most used EWS guides that is developed in UK and distributed around all the world, called National EWS (NEWS) [30].

**Figure 3:**
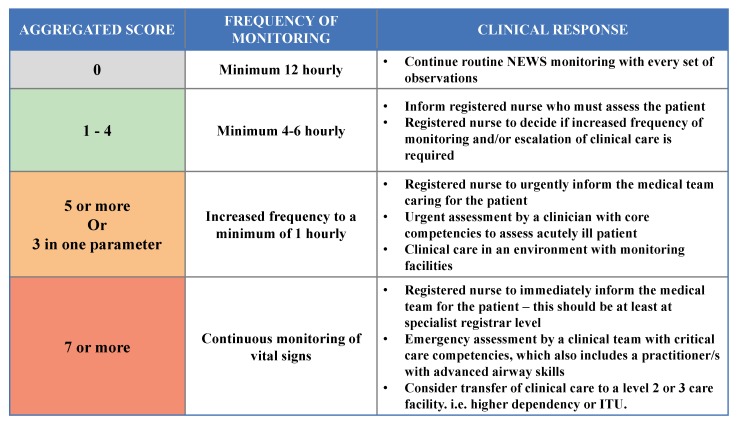
NEWS Clinical Response (NEWS-CR) [30].

Once the score set at each period is calculated, we propose to directly analyze the collected records and alert, ideally using phone device, the medical staff by the appropriate response. In our platform, we are interested in the clinical responses guide proposed in [30] as one of the most used guides around the world. In this guide, the coordinator calculates the aggregated score of records received at each period and then sends the corresponding response to the medical staff according to Figure 3. For instance, if the score set calculated during a period is ${}_{t}^{}S_{V}^{p}=\left[0,2,1,2\right]$, then the aggregated score equals 5 (i.e., 0+2+1+2) and the coordinator sends an alert to medical staff informing that the frequency of patient monitoring should be increased to at least once per hour.

Algorithm 1 describes the emergency detection and clinical response process, which is applied at the coordinator level. The algorithm takes as input the vector of records collected by all biosensors during a period time *t*. Then, it calculates the score for each record followed by the aggregate score (Lines 2–5). Finally, the coordinator sends an alert to the medical team indicating the clinical response that should be taken according to the situation of the patient (Lines 6 and 7).
**Algorithm 1 ** Emergency detection and clinical response algorithm.**Require:** A patient: *p*, Set of biosensors: $B_{V}^{p}$, A period time: *t*, Records collected during *t*: ${}_{t}^{}R_{V}^{p}=\left[r_{1},r_{2},\cdots ,r_{T}\right]$.
**Ensure:** A clinical response.
1:AggScore=0 2:**for** each record $r_{i}\in {}_{t}^{}R_{V}^{p}$ **do**3: calculate score $s_{i}$ of $r_{i}$ according to EWS 4: AggScore += $s_{i}$ 5:**end for** 6:find the corresponding clinical response from NEWS-CR according to AggScore 7:send an alert containing the clinical response to the medical team 


### 5.4. Patient Archiving Algorithm

Patient data archiving is a key operation in hospitals. On the one hand, it allows medical staff to notice the progress of patient situation over time and, on the other hand, it helps professionals to better understand diseases and improve the healthcare quality. Indeed, data collected by the biosensors are vulnerable to loss before reaching the coordinator due to several reasons: (1) a long distance; (2) the congestion due to the overloaded network in the case of dense biosensors deployment; (3) obstacles; and (4) a failure in the biosensor itself. In such cases, the medical team cannot make the right decision about the patient situation or store missing records for a later analysis. Thus, to overcome missing records, a preprocessing of data should be made before any decision or storage process.

In our platform, we benefit from the Python open source libraries which offer a huge number of functions allowing to preprocess the data before any analysis. Some of the most useful libraries dealing with missing values in Python include Pandas, NumPy, and Scikit-Learn. Subsequently, the missing values can be estimated using functions such as replace, isnull, dropna, fillna, and fit_transform, or using prediction functions such as interpolate. In our simulation, we created a script for Spark called MissRec that allows estimating the missing records during a period of time while focusing on two main functions: ExponentialSmoothing and nanmean. After estimating the missing records, the Spark script sends the regenerated records to the HDFS for storage.

## 6. Layer 3: Patient Classification and Disease Diagnosis

After data storage, the process of data analysis is started to better understand the diseases and try to minimize their future effects. The first step of data analysis is to classify patients into clusters where patients in the same cluster have common characteristics (symptoms and situations). The second step is the study of the correlation between vital signs of patients at the same cluster. This can help medical data analytics to understand disease causes and behaviors, and thus avoid false disease diagnosis and find suitable treatments. In this layer, our objective is to propose two data analysis algorithms, one for the patient classification and the other for the disease diagnosis. It is important to notice that both algorithms are implemented on batch processing mode on the Spark cluster node.

### 6.1. Patient Classification Algorithm

Data classification is a well-known approach for most domains. One can find a huge number of data classification algorithms such as K-nearest neighbors, naïve Bayes, decision tree, etc. [31]. However, Kmeans clustering is still the most used classification algorithm because it can be efficiently adapted to a huge number of applications. Unfortunately, Kmeans has a significant negative impact in data latency, especially in applications that produce big amount of data such as sensing-based healthcare. To overcome this problem, we propose a new version of Kmeans, called SKmeans (Stability-based Kmeans), which is strictly dedicated to sensing-based healthcare applications.

To classify similar patients’ situations using SKmeans, we first determine the stability level of each patient during his journey in the hospital (according to his archive), and then we assign the patient to its nearest group of patients’ stability. Derived from ${}_{t}^{}R_{V}^{p}$, we define the subset ${}_{t}^{s}R_{v}^{p}$, which only contains the records of the vital sign v∈V with a score *s* in ${}_{t}^{}R_{V}^{p}$, where s∈[0,3]. Accordingly, ${}_{t}^{s}S_{v}^{p}$ is the subset with scores *s* of ${}_{t}^{}S_{V}^{p}$ for the set of records ${}_{t}^{}R_{V}^{p}$. Thus, ${}_{t}^{}R_{v}^{p}=\cup_{i=0}^{3}\;\;{}_{t}^{i}R_{v}^{p}$ while ${}_{t}^{}S_{v}^{p}=\cup_{i=0}^{3}\;\;{}_{t}^{i}S_{v}^{p}$. Let |X| be the norm zero, i.e., the number of non-zero elements in a set *X*. For the sake of simplicity, let us assume a portion of records collected for two patients *p* and *q* during a period *t* where two record vectors ${}_{t}^{}R_{v}^{p}$ and ${}_{t}^{}R_{v}^{q}$ are stored, respectively, for *p* and *q*. Then, the stability level, noted as stab, of the vital sign *v* of the two patients can be calculated as the overlap between the number of similar records (having the same scores) in ${}_{t}^{}R_{v}^{p}$ and ${}_{t}^{}R_{v}^{q}$, as shown in the following equation:

(1) $$\begin{array}{c} stab\left({}_{t}^{}R_{v}^{p},{}_{t}^{}R_{v}^{q}\right)=\frac{\sum_{k=0}^{3}min(|{}_{t}^{k}R_{v}^{p}\left|,\right|{}_{t}^{k}R_{v}^{q}\left|\right)}{min(|{}_{t}^{}R_{v}^{p}|,|{}_{t}^{}R_{v}^{q}|)}\times 100 \end{array}$$

where $|{}_{t}^{k}R_{v}^{p}|$ means the number of records with score *k* in ${}_{t}^{}R_{v}^{p}$. Therefore, stab ranges between 0 and 100, where 0 means that both patients have similar stable situation and 100 indicates a severely unstable situation for both patients.

Basically, SKmeans allows patients with similar situation progress to be grouped together in order to find patient correlation and understand the behavior of certain diseases. In our platform, patients can be classified according to one or all vital signs depending on the medical needs. For the sake of simplicity, we describe, in the next section, the process of SKmeans based on one patient’s vital signs v∈V but similar process can be adapted to all other vital signs. Similar to traditional Kmeans, the idea of SKmeans is to first define a positive number of clusters (*K*) and then a set of *K* datasets is randomly selected as the initial centroids of the clusters. After that, for each dataset, the distances to all centroids are calculated where the dataset is assigned to the nearest one. At this point, the new centroid is recomputed for each cluster and the process is repeated until the convergence of the algorithm. Obviously, the optimal number of clusters (*K*) can be selected depending on the number of patients in the hospital and their situations. However, compared to traditional Kmeans, SKmeans has two main differences:

- First, patients are grouped according to the scores of records and not the records themselves.
- Second, in each iteration, a patient is assigned to the nearest centroid based on the stability level calculated using Equation (1) and not the Euclidean distance over the whole record vectors as in the traditional Kmeans. This leads to a huge reduction of the cost of calculation.

Algorithm 2 describes our SKmeans adapted to the stability level in order to classify patients according to a vital sign situation. First, it computes the score vector of a record vector for a patient (Lines 1–5). Then, it randomly selects a set of *K* score vectors as the initial centroids of the clusters (Lines 6–8). For every iteration, it computes the stability between each vector score and all cluster centroids and assigns the patient to the nearest stable centroid (Lines 10–17). Finally, the algorithm calculates the new cluster centroids and iterates until there are no longer changes in the cluster memberships.
**Algorithm 2** SKmeans algorithm.**Require:** A set of patients: $P=[P_{1},P_{2},\cdots ,P_{\gamma }]$, A stored at period *t*, A vital sign: v∈V, Set of stored records: ${}_{t}^{}R_{v}^{}=\left[{}_{t}^{}R_{v}^{1},{}_{t}^{}R_{v}^{2},\cdots ,{}_{t}^{}R_{v}^{F}\right]$, Cluster number: *K*.  
**Ensure: ** Set of patient clusters: $C=\{C_{1},C_{2},\cdots ,C_{K}\}$.
1:${}_{t}^{}S_{v}^{}\leftarrow$∅      // ∅ is an empty set  2:**for** i=1toγ **do**
3: calculate ${}_{t}^{}S_{v}^{i}$  4: ${}_{t}^{}S_{v}^{}\leftarrow {}_{t}^{}S_{v}^{}\cup \left\{{}_{t}^{}S_{v}^{i}\right\}$  5:**end for**  6:**for** i=1toK **do**7: randomly choose centroid $c_{i}$ among ${}_{t}^{}S_{v}^{}$ belongs to $C_{j}$  8:**end for** 9:C←∅10:**repeat**11: **for** each set of scores ${}_{t}^{}S_{v}^{i}\in {}_{t}^{}S_{v}^{}$ **do**12:  // calculate the stability between ${}_{t}^{}S_{v}^{i}$ and all centroids   13:  
**for** k=1toK **do**
14:   $st_{k}=stab$ (${}_{t}^{}S_{v}^{i}$, $c_{k}$)   15:  **end for**  16:  Assign ${}_{t}^{}S_{v}^{i}$ to the cluster $C_{k}$ with nearest centroid $c_{k}$ (i.e., $|st_{k}-c_{j*}|\leq |st_{k}-c_{j}|;\;j\in \left\{1,\cdots ,K\right\}$)  17: **end for**  18: Update the centroids of all clusters  19:**until** no more changes in the cluster memberships  20:return *C*


### 6.2. Disease Diagnosis Algorithm

Getting the correct diagnosis is a key for the patient. Otherwise, the consequences of misdiagnosis are most devastating, which may cause obstruction, getting incorrect medications, or even death. Hence, disease diagnosis takes, day after day, an increasing attention from both clinicians and researchers. Indeed, finding a correlation between the variation of vital signs of the same patient is one of the most efficient ways to make a right diagnosis. The idea behind such approach is to link between the variation of vital signs and the symptoms appeared on the patient, thus the mission of determining the disease becomes easy for the clinicians. More formally, if two or more vital signs are strongly correlated for a huge number of patients while the same symptoms have appeared on such patients, then the accuracy of disease diagnosis is increased. In this section, a disease diagnosis technique based on a modified version of association rule mining algorithm is proposed, which is applied at each cluster after patient classification.

As in the traditional algorithm, our adapted version of association rule mining algorithm consists of a two-step process: find frequent scores and generate strong association. However, our version is more dedicated to sensing-based healthcare and takes into account the record sores when finding correlation among vital signs. Assume a cluster contains θ patients, where θ<γ, with their corresponding stored record vectors ${}_{t}^{}S_{v}^{}=\left[{}_{t}^{}S_{v}^{1},{}_{t}^{}S_{v}^{2},\cdots ,{}_{t}^{}S_{v}^{\gamma }\right]$. Then, we describe both steps used in our algorithm as follows:

- Find frequent scores: This step aims to find the most repetitive scores of each vital sign of a patient to meet a certain threshold. We called the threshold as minimum score strength, expressed as μ, which takes a value between 0 and 100. Then, we calculate the strength, str, of each score ${}_{t}^{s}S_{v}^{p}\in {}_{}^{}S_{v}^{p}$, where s∈[0,3], of a vital sign according to the following equation:
  (2) $$str\left({}_{t}^{s}S_{v}^{p}\right)=\frac{|{}_{t}^{s}S_{v}^{p}|}{|{}_{t}^{}S_{v}^{p}|}\times 100$$
  where s∈[0,3]. Therefore, if $str({}_{t}^{s}S_{v}^{p})\geq \mu$, then the score is considered as a strong score. Indeed, if a vital sign contains more than one score, then the vital sign assigned a unique notation according to Table 1:
  At the end of this step, patients with score notations at each cluster are defined in the form of the following matrix (i∈[0,14]):
  $$\begin{array}{cccc} & & \begin{array}{ccccc} HR & SBP & RR & OS & \cdots \; \end{array} & \\ \begin{array}{c} \begin{array}{c} patient\#1= \end{array}\\ patient\#2=\\ \;\vdots \\ patient\#\theta = \end{array} & ( & \begin{array}{ccccc} I_{HR}^{i} & I_{SBP}^{i} & I_{RR}^{i} & I_{OS}^{i} & \cdots \\ I_{HR}^{i} & I_{SBP}^{i} & I_{RR}^{i} & I_{OS}^{i} & \cdots \\ \vdots & \vdots & \vdots & \vdots & \vdots \\ I_{HR}^{i} & I_{SBP}^{i} & I_{RR}^{i} & I_{OS}^{i} & \cdots \end{array} & ) \end{array}$$
- Generate strong association: In this step, we try to find strong correlation rules between vital signs of all patients. By definition, the rules must be provided from frequent patient scores and must satisfy, in addition to the minimum score strength μ, a minimum confidence expressed as ρ. Assume the set of notations is defined as $I_{v}=\left\{I_{v}^{0},I_{v}^{1},\cdots ,I_{v}^{14}\right\}$. Then, our objective is to find the list of mining rules, $M=\{M_{1},M_{2},\cdots ,M_{\delta }\}$, where each $M_{i}\in M$ is in the form of L=>N; *L* and *N* can be one or an intersection of score notations from $I_{v}$. A rule $M_{i}$ has a strong association mining if its support, $sup(M_{i})$, is greater than the minimum support μ and its confidence, $conf(M_{i})$, exceeds the minimum confidence threshold ρ as follows:
  (3) $$sup\;\left(M_{i}\right)=\frac{\left|L\cap N\right|}{\theta }\times 100\geq \mu ,$$
  where |L∩N| is the number of patients, in the above matrix, that contains the combinations of score notations in *L* and *N*.
  (4) $$conf\;\left(M_{i}\right)=\frac{\left|N\right|}{\left|L\right|}\times 100\geq \rho ,$$

Algorithm 3 describes the process of finding the set of strong association rules among $I_{v}$. The process starts by searching all the possible combinations of notations from $I_{v}$, and then finding the list of all association rules (Lines 1–4). After that, we calculate the support and confidence for each rule and a rule is added to the list of strong rules only if its support and confidence are greater than the defined thresholds μ and ρ, respectively (Lines 5–11). Indeed, the obtained rules will allow clinicians to find the correlation between the variation of vital signs of patients. Consequently, in addition to the symptoms appearing in the corresponding patients, they can have a better understanding about a disease and its diagnosis while giving the right patient medication.
**Algorithm 3** Association mining rules algorithm.**Require:** Set of notations: $I_{v}=\left\{I_{v}^{0},I_{v}^{1},\cdots ,I_{v}^{14}\right\}$, Minimum score strength: μ, Minimum confidence threshold: ρ.
**Ensure:** Set of strong mining association rules: *M*.
1:find all combinations of notations, *T*, from $I_{v}$ 2:$T\leftarrow \left\{I_{v}^{i},\mathrm{where}\;i\in \left[0,14\right]\right\}\cup \left\{I_{v}^{i}\cap I_{v}^{j},\mathrm{where}\;i,j\in \left[0,14\right]\;\mathrm{and}\;i\neq j\right\}\cup \cdots \cup I_{v}$ 3:find all combinations of mining rules, *U* 4:U={L=>N,whereLandN∈T} 5:M←∅6:**for** each rule $U_{i}\in U$ **do**7: calculate $sup\;(U_{i})$ and $conf\;(U_{i})$ 8: **if** $sup\;(U_{i})\geq \mu$ and $conf\;(U_{i})\geq \rho$ **then**9:  $M\leftarrow M\cup \{U_{i}\}$ 10: **end if** 11:**end for** 12:**return** *M*


## 7. Layer 4: Data Retrieval and Visualization

The last layer of our platform is dedicated to allow medical staff access to the patient records stored in the Hadoop HDFS. It relies on two data retrieval tools (Hive and Spark SQL) and one graphing tool (Matplotlib). On the one hand, data retrieval tools are responsible for obtaining data from the Hadoop storage system using a set of criteria defining via queries. The retrieved data are mostly stored in a file or displayed on the screen. On the other hand, data visualization is simply a graphical representation of the retrieved data using statistical graphics or plots. In the next sections, we highlight each tool used in our platform.

### 7.1. Hive

Hive [28] is software used for data warehousing implemented on top of Hadoop HDFS in order to provide data query and analysis. In addition, Hive allows creating a metadata storage in the form of tables in a relational database system. This makes our platform more efficient in terms of reducing the access time to the patient archive. On the other hand, it helps the medical staff to keep track of the criticality of patients in real-time. In our platform, we installed Hive on the cluster master node and we created an external table located in the HDFS main directory, where the medical staff can explore the data imported to HDFS using HiveQL console. To retrieve the data, they have to write HiveQL queries depending on their requirements. For instance, they can ask for all patient IDs having critical HR records as follows:

SELECT patient_id FROM Patient WHERE record <51 or record >90 

### 7.2. Spark SQL

Spark SOL [25] is a component that can be installed on top of Spark core and aims to provide a data abstraction called DataFrames to handle structured database. DataFrames is usually handled using a specific language named domain-specific language (DSL) [25], which is manipulated using Python in our platform. After installing it on the master node, the medical staff can run the Spark SQL console using the command *spark-sql* to access the patient data on Hadoop storage. Then, they have to use HiveQL syntax, which is supported by Spark SQL to make various queries.

### 7.3. Matplotlib

Matplotlib [29] is the most used plotting library in Python programming with its extension NumPy. In our platform, we created a Python script that allows reading periodic patient data sent to Hadoop storage and visualizing them, using Matplotlib, on a real-time graph to the medical staff. An important extension which can be added later to our platform is to send plotting graphs to the clinicians using mobile application, so that they can monitor the patient situation far from the hospital.

## 8. System Demonstration and Evaluation

To evaluate its performance, we developed our platform based on two phases: Hadoop installation and algorithms’ implementation. On the one hand, we installed Hadoop 3.0.1 on a physical machine with an i7 processor, 8 CPUs 2.70 GHz, and 16 GB of memory. Then, we created a four-node Hadoop cluster on four virtual machines with Ubuntu 18.04 as an operating system. In addition, a virtual machine was configured to act as the master node and the three other machines were configured to act as slave nodes. We installed all layer tools (ingestion, processing, and visualization) on the master node while the slave nodes were responsible for storing patient data. On the other hand, we used real health data collected from MultipleIntelligent Monitoring in Intensive Care (MIMIC) database of PhysioNet [32]. MIMIC contains data for about 72 patients where records about vital signs include Heart Rate (HR), Systolic Blood Pressure (SBP), Respiration Rate (RR), and Oxygen Saturation (OS). Every second, the biosensor collects new reading for each vital sign, and then it sends toward the Hadoop cluster for processing and archiving purposes. In our simulation, we used a file that includes a log of about 100,000 readings for each patient. To make our simulation more realistic, patient records are stored separately in a file in the main directory of the master node. Then, we created Java and Python scripts that allow reading data for each patient while applying one of the proposed algorithms.

Table 2 summarizes the parameters used in our platform with their tested values.

### 8.1. Records Patients Study

**Figure 4:**
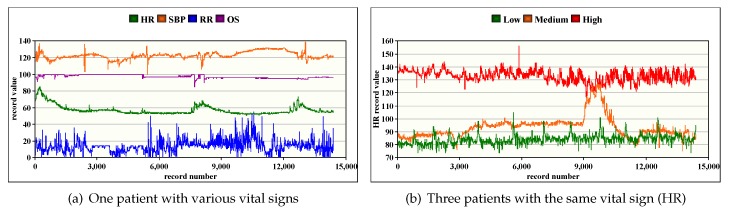
Variation of raw record data during 4 h of patient monitoring.

In this section, we show the patient situation progress after storing data in the HDFS which can help in understanding the stability level of each patient. In Figure 4a, we show a portion of 15,000 record values (e.g., 4 h) for each vital sign of a patient while, in Figure 4b, we only show HR records for three patients with different criticality situations, namely low, medium, and high. The obtained results reveal many observations: First, the stored records for each vital sign of a patient are highly redundant. Second, we can also observe that some vital signs are correlated where the variation/stability of one can influence the others (Figure 4a). Third, we notice that low and high criticality patient situations produce more data redundancy than a patient with medium situation (Figure 4b).

### 8.2. SKmeans Study

**Figure 5:**
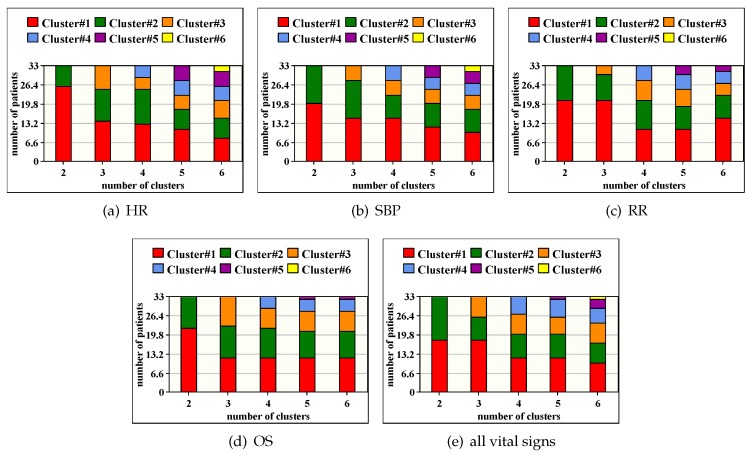
Distribution of patients over clusters.

Our objective is to study the SKmeans algorithm proposed at the third layer in our platform for patients’ classification. As mentioned above, classification can be done according to one or all vital signs of the patients depending on the medical staff decision. Figure 5 shows the distribution of patients over clusters after applying SKmeans algorithm when varying *K* from 2 to 6. We can notice several observations according to the obtained results: First, the patients are not equally distributed into clusters, which confirms the behavior of SKmeans of classifying patients based on their stability level and not in an equal way. Second, clusters approximately have the same number of patients regardless of the monitored vital sign (Figure 5a–d). This confirms the existence of a correlation between the variation of vital signs of the same patient. Third, we obtain similar patients’ classification when applying SKmeans on one vital sign (Figure 5a–d) and all vital signs (Figure 5e). Fourth, we notice that the number of clusters converges to 5 with RR and OS biosensors.

**Figure 6:**
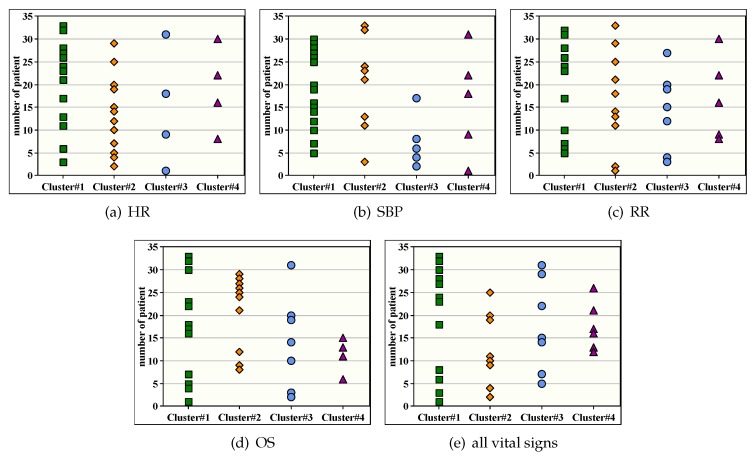
Illustrative example for distribution of patients’ IDs over clusters.

According to Figure 5, Figure 6 shows a distribution map for the patient IDs classification over the clusters after applying SKmeans. The objective of this figure is to verify if patients with similar stability are always assigned to the same cluster for various vital signs or not. The number of clusters is fixed to 4. As expected, the obtained results show a significant correlation between vital signs where patients with same records’ variation are grouped together. Therefore, we consider SKmeans as an efficient clustering algorithm for sensing-based healthcare application in terms of classifying patients according to their stability level and their situation progress.

### 8.3. Iteration Number Study

**Figure 7:**
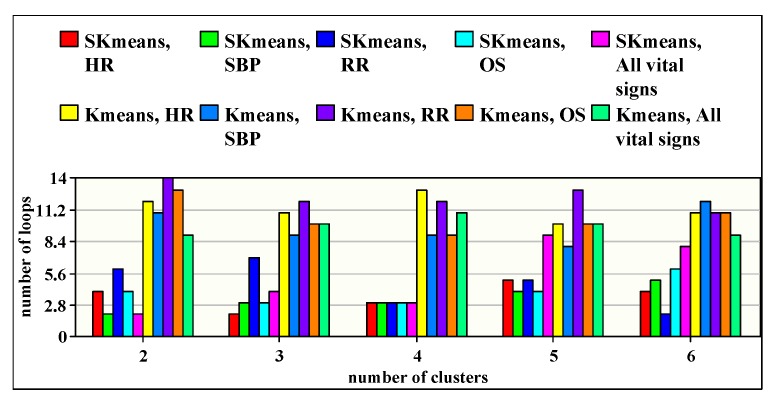
Number of iterations when applying SKmeans and traditional Kmeans.

In Figure 7, we show the iteration number required by SKmeans to find the final clusters of patients, compared to those obtained with traditional Kmeans. Indeed, this factor becomes important to study in sensing-based healthcare since it affects the latency of decision making which is a critical factor in the study of emergency situations. The iteration number is mostly related to initial centroids which are randomly selected in both SKmeans and Kmeans. The obtained results show that SKmeans outperforms Kmeans in terms of minimizing the number of iterations with all vital sign conditions. Subsequently, the number of iterations in SKmeans varies from 2, in the worst case, to 9, in the best case, and from 8 to 14 using Kmeans. Therefore, SKmeans ensures a fast computation process to the medical staff.

### 8.4. Processing Speed Study

**Figure 8:**
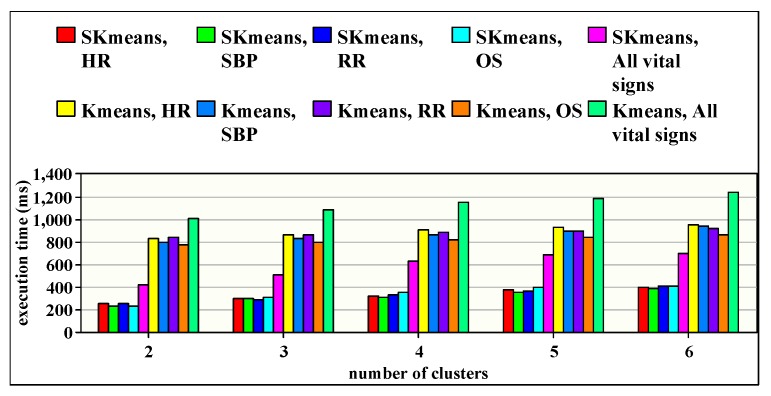
Execution time when applying SKmeans and Kmeans.

Figure 8 shows the processing speed, or the execution time, required to apply SKmeans and Kmeans algorithms for the various vital signs. Mostly, the processing speed is an indicator to the robustness of any system. In our platform, the processing speed is highly dependent on the iteration numbers shown in Figure 7; the greater is the iteration number, the longer is the processing time required and the lower is the robustness. Since SKmeans algorithm highly reduces the number of iterations, it simultaneously reduces the execution time compared to the traditional Kmeans. Subsequently, SKmeans can optimize the processing time speed up to three times compared to Kmeans.

### 8.5. Clustering Accuracy Study

Clustering accuracy is one of the most important metric in order to evaluate the performance of the formed final clusters. Indeed, one can find various coefficients that is proposed in the literature to evaluate the clustering. In this paper, we select the Silhouette coefficient [33] as one of the most popular metrics to evaluate the accuracy of SKmeans and Kmeans algorithms. By definition, the Silhouette coefficient score ranges between −1 (which indicates a low clustering accuracy) and +1 (which indicates a high clustering accuracy) while 0 indicates nested clusters. Generally, the Silhouette coefficient is a composite index that indicates the cohesion and separation of the clusters and can apply several distance measures. In this work, the Silhouette coefficient is adapted to the Euclidean distance that is calculated as follows:

(5) $$S=\frac{E_{i}\left({}_{t}^{}R_{V}^{p}\right)-E_{j}\left({}_{t}^{}R_{V}^{p}\right)}{max(E_{i}\left({}_{t}^{}R_{V}^{p}\right),E_{j}\left({}_{t}^{}R_{V}^{p}\right))}$$

where $E_{i}\left({}_{t}^{}R_{V}^{p}\right)$ is the mean Euclidean distance of the record set ${}_{t}^{}R_{V}^{p}$ to other record sets within the same cluster and $E_{j}\left({}_{t}^{}R_{V}^{p}\right)$ is the mean Euclidean of record set ${}_{t}^{}R_{V}^{p}$ to record sets in the other clusters.

**Figure 9:**
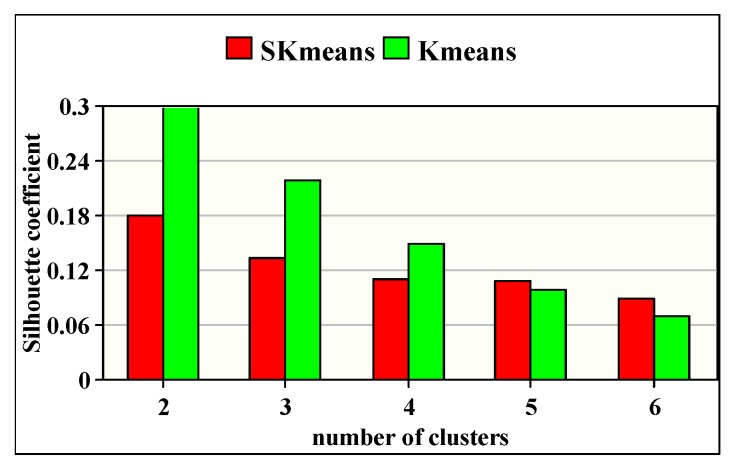
Clustering accuracy of SKmeans and Kmeans.

Figure 9 shows the Silhouette coefficient of the heart rate vital sign when varying the number of clusters *K* from 2 to 6. The obtained results show that both algorithms (SKmeans and Kmeans) give important results regarding the clustering accuracy for various number of clusters. Subsequently, the clustering accuracy obtained with SKmeans varies between 0.09 and 0.18 while it varies between 0.07 and 0.3 using Kmeans. We can also observe that the clustering accuracy decreases with the increasing of the number of clusters.

### 8.6. Vital Signs and Disease Diagnosis Study

**Figure 10:**
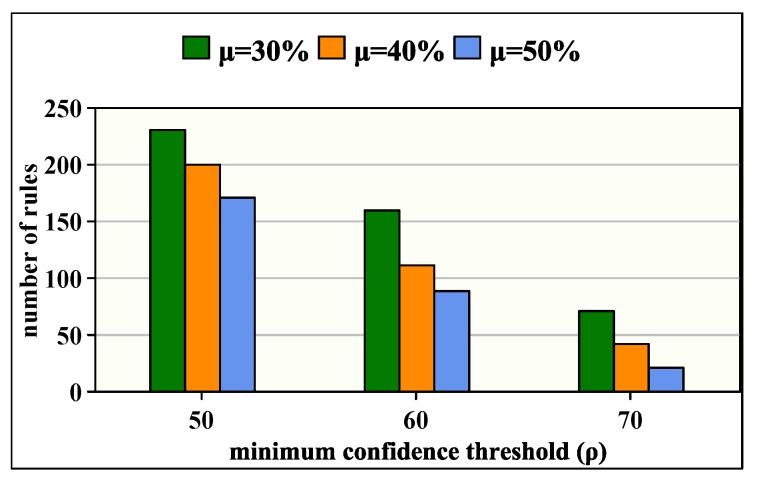
Variation of number of rules as a function of μ and ρ.

Figure 10 shows the number of mining rules generated after applying association rule algorithm adapted to sensing-based healthcare. Indeed, these rules allow finding correlations between vital signs and thus help the medical staff to understand the disease behavior. The obtained results depend on the minimum support threshold (μ) and the minimum confidence threshold (ρ). As shown, our algorithm allows generating many rules—between 21 and 232—for various values of μ and ρ. Subsequently, we also observe that the number of rules decreases when increasing μ or ρ values; however, this makes the rules stronger and more accurate.

### 8.7. Further Discussions

In this section, we give further consideration to the proposed SKmeans algorithm by discussing and analyzing its performance, regarding several metrics, under various conditions and circumstances of the application.

From the processing speed point of view, SKmeans can highly reduce the execution time required for the clustering process compared to the traditional Kmeans. This is due to two reasons: first, the fewer iterations required for the convergence of the SKmeans algorithm (see results on Figure 7), and, second, SKmeans assigns the patients into clusters according to their criticality level, that is calculated once during all iterations, which reduces the processing time overhead of the clustering (see results in Figure 8). Therefore, SKmeans can ensure a fast patient clustering technique, which is required in healthcare applications, especially in emergency cases.

From the accuracy point of view, both SKmeans and traditional Kmeans ensure an acceptable level of clustering accuracy for the healthcare applications. However, the random selection of the cluster centroids considered in both SKmeans and Kmeans should be optimized to increase their performance. Furthermore, in the healthcare cases where most of the patients have stable situations, SKmeans can ensure an accurate data clustering; otherwise, the obtained clustering accuracy decreases.

## 9. Conclusions and Future Work

The investment in sensing-based healthcare applications continues to rise in this decade as the public health attracts more attention day by day from governments and industries. In this paper, we propose an efficient and robust big data analytics platform for real-time patient monitoring and assessment. The architecture of our platform is mainly based on Hadoop ecosystems and it consists of four layers: real time patient monitoring, real time decision and data storage, patient classification and disease diagnosis, and data retrieval and visualization. In addition, our platform includes various data analytical algorithms for each layer in order to make patient classification and help to find correlations between variable of vital signs and diseases. We demonstrated the relevance of our platform based on real heath data according to several parameters.

As future work, we have three main directions to enhance our platform. First, we plan to test our platform in real-case scenarios in order to validate its performance. Second, we seek to adapt our platform to take into account various types of patient data such as images for organs, video for operations, etc. Finally, we plan to develop a mobile application in order to help clinicians closely and remotely monitor critical patients.

## Figures and Tables

**Table 1:** Notation of scores.

Strong Scores	Notation
${}_{t}^{0}S_{v}^{p}$	$I_{v}^{0}$
${}_{t}^{1}S_{v}^{p}$	$I_{v}^{1}$
${}_{t}^{2}S_{v}^{p}$	$I_{v}^{2}$
${}_{t}^{3}S_{v}^{p}$	$I_{v}^{3}$
${}_{t}^{0}S_{v}^{p}$ and ${}_{t}^{1}S_{v}^{p}$	$I_{v}^{4}$
${}_{t}^{0}S_{v}^{p}$ and ${}_{t}^{2}S_{v}^{p}$	$I_{v}^{5}$
${}_{t}^{0}S_{v}^{p}$ and ${}_{t}^{3}S_{v}^{p}$	$I_{v}^{6}$
${}_{t}^{1}S_{v}^{p}$ and ${}_{t}^{2}S_{v}^{p}$	$I_{v}^{7}$
${}_{t}^{1}S_{v}^{p}$ and ${}_{t}^{3}S_{v}^{p}$	$I_{v}^{8}$
${}_{t}^{2}S_{v}^{p}$ and ${}_{t}^{3}S_{v}^{p}$	$I_{v}^{9}$
${}_{t}^{0}S_{v}^{p}$ and ${}_{t}^{1}S_{v}^{p}$ and ${}_{t}^{2}S_{v}^{p}$	$I_{v}^{10}$
${}_{t}^{0}S_{v}^{p}$ and ${}_{t}^{1}S_{v}^{p}$ and ${}_{t}^{3}S_{v}^{p}$	$I_{v}^{11}$
${}_{t}^{0}S_{v}^{p}$ and ${}_{t}^{2}S_{v}^{p}$ and ${}_{t}^{3}S_{v}^{p}$	$I_{v}^{12}$
${}_{t}^{1}S_{v}^{p}$ and ${}_{t}^{2}S_{v}^{p}$ and ${}_{t}^{3}S_{v}^{p}$	$I_{v}^{13}$
${}_{t}^{0}S_{v}^{p}$ and ${}_{t}^{1}S_{v}^{p}$ and ${}_{t}^{2}S_{v}^{p}$ and ${}_{t}^{3}S_{v}^{p}$	$I_{v}^{14}$

**Table 2:** Simulation environment.

Parameter	Symbol	Values
number of patients	F	72
number of features	T	[HR, SBP, RR, OS]
number of clusters	*K*	2, 3, 4, 5, 6
minimum score strength	μ	30%, 40%, 50%
minimum score confidence	ρ	50%, 60%, 70%
